# The Fallacy of the Theoretical Meaning of Formative Constructs

**DOI:** 10.3389/fpsyg.2018.00179

**Published:** 2018-02-15

**Authors:** Hervé Guyon

**Affiliations:** ^1^Sceaux IUT (University Institute of Technology), Université Paris-Sud, Orsay, France; ^2^UMR6308 Aménagement des Usages des Ressources et des Espaces Marins et Littoraux, Plouzane, France; ^3^Centre for Research in Epidemiology and Population Health (Institut National de la Santé et de la Recherche Médicale), Villejuif, France

**Keywords:** epistemology, ontology, formative measurement, theoretical meaning, empirical meaning

## Introduction

The gap between advanced statistical approaches and epistemological discussion is certainly one of the most important problems in psychometrics to really improve psychological measures. An important issue in measurement models in social science is the debate about formative measurement models. For some years, the debate has been essentially focused on methodological issues. We aim in this short article to contribute to a critical discussion of formative models based on ontological discussion (*theoretical meaning*).

The mainstream in psychology is based on empirical realism (see: Slaney, 2001; Maraun and, 2013). This position holds that psychological attributes are realities that we seek to characterize independently from the knower, as in physics. Clearly all the discussion about formative measurement models is, more or less consciously, based on this empirical realism epistemology. In opposition to empirical realism, a pragmatist current is emerging and poses that the ontology of psychological attributes is not independent from social praxis (Maul, 2013; Allen and Clough, 2015; Maul et al., 2016; Guyon et al., 2017; Maul and McCrae, 2017). On the basis of this pragmatism epistemology, we argue that we should stop using formative measurement models, not only because of empirical issues but also for theoretical reasons.

### Psychological attributes

There is active discussion about the ontology of psychological attributes. Psychological attributes are psychological properties of an individual (Markus and Bosom, 2013).

As Searle (1996) or Hacking (2000) propose, psychological attributes do not exist as entities independent from human perceptions. But they do correspond to some kind of reality. This “in-between” position between empirical realism and constructivism (which considers psychological attributes to be a useful fiction), could be related to a new-pragmatism epistemology (Maul, 2013; Allen and Clough, 2015; Guyon et al., 2017; Maul and McCrae, 2017). A psychological attribute is a real object because a psychological attribute derives from the activity of the brain, which we can call *mental processes* in the sense of physical-chemical processes occurring in the brain. It is therefore possible to consider that the instantiation of the psychological attribute exists spatiotemporally, but psychological attributes “*do not correspond to brain organization in a one-to-one fashion*” (Barrett, 2009, p. 328). According to Zachary (2010) or Hare (2016), psychology should break with the dominant epistemology of “biological realism” (Lloyd, 2010), which considers a psychological attribute as a specific physical entity in the brain. A psychological attribute could be considered as an *emergent property* arising within the system of mental processes because of the new structures and functions that emerge from mental processes (Humphreys, 2008; Barrett, 2009; Fingelkurts et al., 2013; Maul, 2013; Guyon et al., 2017). The emergent property is not only a novel structure, it is also conceptually novel because we cannot describe the psychological attribute by the concepts used to describe the mental processes (Humphreys, 2008; Barrett, 2009, 2011; Fingelkurts et al., 2013; Maul, 2013). A psychological attribute is in consequence not reducible to mental processes in a predictable or non-reductionist way (Barrett, 2009; Maul, 2013).

The reality (and conceptualization) of a psychological attribute resides in its functional appearance and is derived from human experience and social interactions (Hacking, 2000; Maul, 2013; Guyon et al., 2017). The categories used in psychology (psychological concepts) are observer-dependent (Searle, 1996). Psychological attributes are classified according to their manifestations and their function in social communication (Barrett, 2009). These categories are linked to physical realities, but physical reality is not only what is in the brain, because physical reality also entails social interaction (Thompson and Varela, 2001; Hare, 2016). A concept in psychology can thus be considered as referring neither to a fixed reality (external from social praxis), nor to a singular construction independent from physical reality (Maul, 2013). The categories used in psychology are relational entities, interactive genres (Hacking, 2000).

This pragmatist epistemology is not a radical relativist or constructivist position, because there is a material reality of a psychological attribute, but it is “*a new pattern of reality*” (Fingelkurts et al., 2013, p. 5) that can only be understood as a holistic reality formed by inter-subjective interaction (Varela et al., 1992; Thompson and Varela, 2001; Fingelkurts et al., 2013; Guyon et al., 2017). This is not a criticism of neurobiology, but neurobiology and experimental psychology are in two incommensurable paradigms in the sense given by Kuhn or Feyerabend.

### Empirical meaning and theoretical meaning of formative measurement models

The core of the discussion on formative measurement models has been the “empirical meaning” of such measures. With a few exceptions (Bollen and Diamantopoulos, 2017), most authors consider now that formative measurement is irrelevant because the empirical meaning is a fallacy (Edwards, 2011; Rhemtulla et al., 2015; Aguirre-Urreta et al., 2016a; Bentler, 2016; Cadogan and Lee, 2016; Guyon and Tensaout, 2016; Howell and Breivik, 2016; Lee and Chamberlain, 2016; Markus, 2016; Hardin, 2017). But the academic literature seems to consider the idea that “*constructs themselves, posited under a realist philosophy of science as existing apart from their measurement, are neither formative nor reflective*” (Wilcox et al., 2008, p. 1220) as providing “evidence.” Bagozzi (2011) for example proposed a synthesis on construct measurement and considered that the theoretical meaning of a formative construct does not differ in nature from a reflective construct; their meanings diverge only on the empirical level. Similarly Hardin (2017, p. 598) wrote: “*Constructs exist independently from their measures; theory determines whether indicators cause or measure latent variables*.” Aguirre-Urreta et al. (2016a, p. 77) likewise stated: “*Constructs are concepts whose meaning is provided by the researcher as part of the conceptualization process, which precedes any considerations of how the construct is to be measured*.”

Burt (1976) was the first to highlight the significance of distinguishing between the nominal and the empirical meaning of a construct. The nominal meaning is the interpretation initially postulated by the practitioner. On the basis of “auxiliary theory” (Sajtos and Magyar, 2016), the empirical meaning is that obtained once the measurement model used to represent the construct has been assessed. When these two meanings differ, the specified measures are then subject to interpretational confounding, which appears as the core of the problem with causal indicators (Howell et al., 2007; Aguirre-Urreta et al., 2016a). But how should a construct represented by causal indicators be apprehended theoretically? With a formative measure, we do not observe manifestations of a real (or supposedly real) entity, we hypothesize that an unobservable entity is defined by “causal indicators.”

### The ontological status of psychological attributes measured formatively

**Figure 1:**
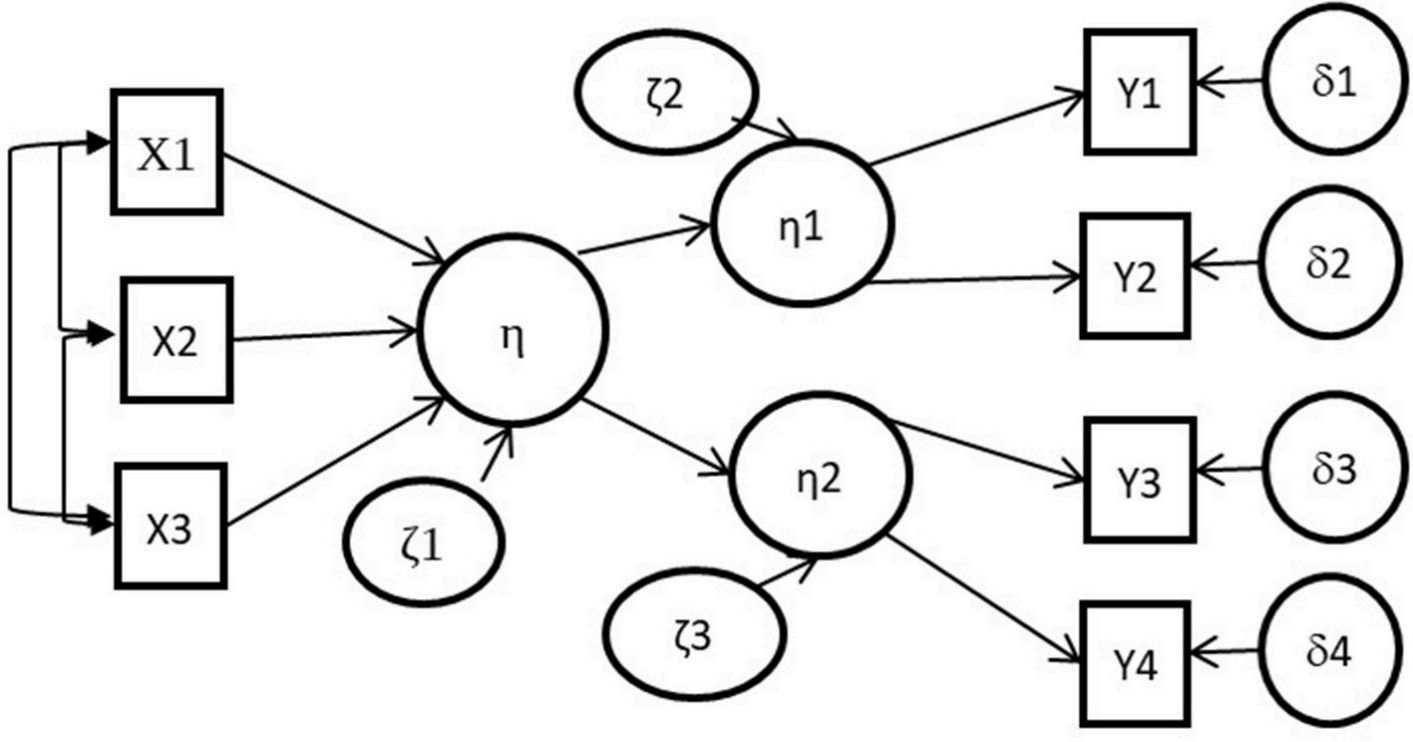
A formative latent variable links to two reflective latent variables.

MIMIC models are rarely used in applied research (Aguirre-Urreta et al., 2016b). Thus, formative constructs discussed here have no manifest variables, but only causal indicators. A formative construct of this sort cannot be modeled without affecting other reflexive latent variables (Figure 1).

It should be noted that *eta* must refer to a real entity and cannot be considered as a pure construction. If *eta* is prespecified by the researcher (as a non-realist entity), *eta* cannot have an error term (kis1), and becomes an *index* (Bollen, 2011). As Bollen and Diamantopoulos (2017) recalled: a latent variable related to a real entity with an error term of zero can occur under certain special circumstances, but this is however extremely unlikely in practice (Bollen and Diamantopoulos, 2017).

The central question discussed here is the theoretical legitimacy of *eta* in the model (Figure 1). In Figure 1, both *eta1* and *eta2* refer to psychological attributes that have theoretical meaning because they represent (in the statistical model) psychological attributes that have observable manifestations (represented by the Ys). But *eta* (the formative latent variable) relates to an entity that has no observable manifestation. This is the ontological issue.

A psychological attribute is a psychological property of an individual, and it can be an entity that exists only in the social space. A psychological attribute exists if and only if it has perceptible manifestations; if not, nobody can consider that a particular attribute characterizes a specific person. A psychological attribute must therefore have manifestations in order to be “real” in our social praxis: “*the appearance is the reality*” (Maul, 2013, p. 757). The conceptualization, and therefore the measurement approach, must start from the perceptible manifestations of the psychological attribute, which underpin the theoretical meaning. In consequence, only a reflective measurement model based on perceptible manifestations can be used to link a latent variable (empirical meaning) to a concept (theoretical meaning) (Guyon et al., 2017).

The hypothetical entity referred to as *eta* has no perceptible manifestations in the Figure 1. Because this entity is linked to the psychological attributes represented by *eta1* and *eta2*, we must consider that the entity represented by *eta* is an entity underpinning psychological attributes: an entity in the brain that generates other psychological attributes which in turn generate perceptible manifestations. Except for very rare psychological attributes, entities in the brain underpinning psychological attributes are not fixed entities. So, *eta* relates to mental processes, physical-chemical processes underpinning psychological attributes. *Eta1* and *eta2* are emergent properties. In a linear model we cannot link *eta* (mental processes) to *eta1* and *eta2* (psychological attributes) because the psychological attributes that emerge from mental processes are not reducible to the mental processes in a predictable or non-reductionist way. These are incommensurable paradigms: mental processes are what is in the brain, psychological attributes are what is in the social space. This is the reason why, generally, models with psychological attributes do not introduce “mental processes” into the models, but tend only to model (linear) relations between psychological attributes. Concretely, in the overall model (Figure 1) *eta* relates to a psychological attribute in a realist epistemology, and at the same time *eta* cannot relate to a psychological attribute. So we need to remove *eta* from the model.

Common examples of formative measurement models are “exposure to discrimination” and “Socio-Economic Status” (SES). It may be that a “perception of socio-economic status” or a “feeling of discrimination” (or “belief in one's socio-economic status” or “belief in discrimination”) can be experienced by individuals, and that these psychological attributes could generate perceptible manifestations. Clearly, a “feeling of discrimination” or a “perception of Socio-Economic Status” are not the same objects as actual “exposure to discrimination” or “Socio-Economic Status.” We are not saying that there is no reality in “exposure to discrimination” or “Socio-Economic Status,” and they can therefore be conceptualized; but they are not psychological attributes because they have no perceptible manifestations conceptually linked to these constructs. Certain social or personal characteristics could generate certain psychological characteristics in an individual, but as Lee and Chamberlain (2016) recalled, causal indicators do not determine the meaning of psychological attributes, they are only their causes. These sociological concepts are not psychological characteristics of a person (as a “perception of socio-economic status” or a “feeling of discrimination” can be), and they can be formalized in a statistical model, on the basis of causal indicators, using an *index* (without error term *ksi1* in Figure 1), but not using a latent variable.

## Conclusion

Widaman (2014) considers that a formatively measured psychological attribute can be considered as an “emergence” of its indicators. We consider that psychological attributes are emergent properties of an individual. But an emergent property is a reality because it exists through empirical manifestations. The emergent property of a formative construct referred to by Widaman has no empirical manifestations (*eta1* and *eta2* in Figure 1 are not empirical manifestations), and so we consider that this use of the emergence concept is in this instance an explanatory artifice. Psychological attributes are derived from human experience; the concept (theoretical meaning) results from perceptible manifestations of an emergent property (the psychological attribute) and therefore the empirical meaning (latent variable) must necessarily be linked to a reflective measurement model. Our position does not deny that there can be exogenous factors (causal indicators) that could influence psychological attributes, but these exogenous factors cannot be considered as providing the theoretical meaning of a psychological attribute. Only perceptible (reflexive) manifestations can drive the meaning of a psychological attribute.

## Author contributions

The author confirms being the sole contributor of this work and approved it for publication.

### Conflict of interest statement

The author declares that the research was conducted in the absence of any commercial or financial relationships that could be construed as a potential conflict of interest.
